# Decoding Primary Hyperlipoproteinemias: A Focus on the Pathogenesis and Diagnosis of Familial Hypercholesterolemia and Familial Combined Hyperlipidemia

**DOI:** 10.3390/diagnostics16152313

**Published:** 2026-07-23

**Authors:** Iris Bararu-Bojan, Maria Cristina Vladeanu, Dan Iliescu-Halitchi, Carmen Elena Plesoianu, Andrei Bojan, Otilia Elena Frasinariu, Razvan Cosmin Tudor, Manuela Ciocoiu, Catalina Tudor, Codruta Iliescu-Hailitchi, Amin Bazyani, Cezar Ilie Foia, Oana-Viola Badulescu

**Affiliations:** 1 Department of Morpho Functional Sciences, Faculty of Medicine, Grigore T. Popa University of Medicine and Pharmacy, 16 Universitatii Street, 700115 Iasi, Romania; iris.bararu@umfiasi.ro (I.B.-B.);; 2 Department of Internal Medicine II, Faculty of Medicine, Grigore T. Popa University of Medicine and Pharmacy, 16 Universitatii Street, 700115 Iasi, Romania; 3 Department of Surgical Sciences, Faculty of Medicine, Grigore T. Popa University of Medicine and Pharmacy, 16 Universitatii Street, 700115 Iasi, Romania; 4 Department of Pediatrics, Faculty of Medicine, Grigore T. Popa University of Medicine and Pharmacy, 16 Universitatii Street, 700115 Iasi, Romania; 5 Heart Institute GIM Georgescu Iasi, 700503 Iasi, Romania

**Keywords:** primary hyperlipoproteinemias, familial hypercholesterolemia, familial combined hyperlipidemia

## Abstract

Primary hyperlipoproteinemias represent a heterogeneous group of inherited lipid metabolism disorders characterized by persistent abnormalities in plasma lipoproteins, a markedly increased risk of premature atherosclerotic cardiovascular disease (ASCVD) and, in selected phenotypes, acute pancreatitis. Traditionally classified according to the Fredrickson phenotypic system, these disorders are now increasingly understood through a multidimensional framework integrating molecular genetics, intracellular lipid trafficking, inflammatory signaling, and systemic metabolic regulation. Recent advances have identified both monogenic and polygenic determinants underlying disease expression, including pathogenic variants affecting LDLR, APOB, PCSK9, APOE, and lipoprotein lipase pathways, as well as the cumulative contribution of multiple common lipid-associated variants. Furthermore, emerging evidence highlights the role of endoplasmic reticulum stress, oxidative imbalance, adipose–hepatic crosstalk, intestinal lipid absorption, and inflammatory mediators in modulating lipoprotein metabolism and cardiovascular risk. Novel regulators such as angiopoietin-like proteins (ANGPTLs), microRNAs, and pathways involved in cholesterol efflux and remnant lipoprotein clearance have further refined our understanding of disease heterogeneity and therapeutic responsiveness. Familial hypercholesterolemia and familial combined hyperlipidemia exemplify the complex interplay between genetic susceptibility, metabolic dysfunction, and environmental influences that shape phenotype severity and long-term cardiovascular outcomes. Advances in diagnostic strategies, including genetic testing, polygenic risk scores, apolipoprotein profiling, and vascular imaging, have significantly improved risk stratification and personalized management. Simultaneously, innovative therapies—including PCSK9 inhibitors, ANGPTL3-targeted agents, antisense oligonucleotides, and RNA-silencing technologies—are reshaping treatment paradigms and expanding options for high-risk patients. This chapter synthesizes contemporary insights into the pathogenesis of primary hyperlipoproteinemias, emphasizing the transition from traditional lipid-based classification toward precision medicine approaches focused on lifetime cardiovascular risk, molecular characterization, and individualized therapeutic intervention.

## 1. Introduction

**Figure 1:**
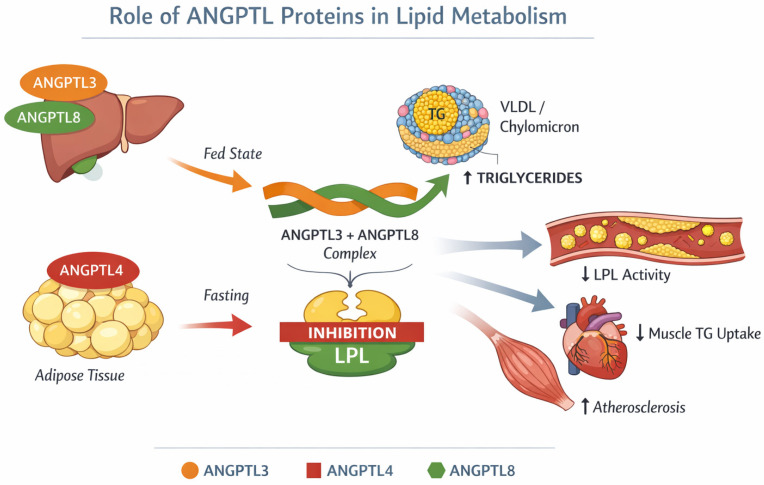
Role of ANGPTLs in lipidic metabolism.

Primary hyperlipoproteinemias comprise a heterogeneous group of inherited disorders of lipoprotein metabolism characterized by persistent abnormalities in plasma lipid and lipoprotein levels, a markedly increased risk of atherosclerotic cardiovascular disease and, in selected phenotypes, acute pancreatitis. Traditionally classified according to the Fredrickson phenotypic system, these disorders are now increasingly understood through a molecular and pathophysiological lens that integrates genetics, lipid trafficking, and systemic metabolic regulation. Recent advances have revealed that primary hyperlipoproteinemias arise not only from monogenic defects affecting key enzymes, receptors, or apolipoproteins—such as lipoprotein lipase, LDL receptor, apolipoprotein B, or PCSK9—but also from complex polygenic architectures interacting with environmental and epigenetic factors. Emerging data highlight the pivotal role of intracellular lipid handling, endoplasmic reticulum stress, inflammatory signaling, and dysregulated crosstalk between hepatocytes, adipose tissue, and the intestine in disease initiation and progression. In addition, novel regulators of lipoprotein metabolism, including ANGPTLs, microRNAs, and pathways governing cholesterol efflux and remnant clearance, have refined our understanding of disease heterogeneity and clinical expression (Figure 1). This chapter synthesizes current insights into the pathogenesis of primary hyperlipoproteinemias, bridging classical concepts with contemporary molecular discoveries, and discusses their implications for refined phenotyping, risk stratification, and the development of targeted therapeutic strategies [1,2].

## 2. Materials and Methods

This narrative review was conducted to synthesize current evidence on the molecular mechanisms, genetic determinants, inflammatory pathways, diagnostic strategies, and emerging therapeutic perspectives related primarily to familial hypercholesterolemia and familial combined hyperlipidemia.

A comprehensive literature search was performed in PubMed/MEDLINE, Scopus, Web of Science, and Google Scholar for articles published in English between January 2000 and March 2026. The final literature search was conducted in April 2026. Landmark articles published before 2000 were also considered when they were deemed historically or scientifically relevant to the understanding of inherited lipid disorders.

The search strategy combined Medical Subject Headings and free-text keywords related to inherited dyslipidemias and lipoprotein metabolism. The principal terms included “primary hyperlipoproteinemia,” “familial hypercholesterolemia,” “familial combined hyperlipidemia,” “dyslipidemia genetics,” “lipoprotein metabolism,” “LDLR,” “APOB,” “PCSK9,” “ANGPTL3,” “lipoprotein(a),” “polygenic hypercholesterolemia,” “endoplasmic reticulum stress,” “oxidative stress,” “microRNA,” “intestinal cholesterol absorption,” “inflammatory pathways,” “atherosclerosis,” “precision medicine,” and “lipid-lowering therapy.” Boolean operators “AND” and “OR” were used to combine the search terms according to the requirements of each database.

Eligible publications included original research articles, systematic reviews, meta-analyses, consensus statements, clinical guidelines, and translational studies addressing the pathogenesis, molecular biology, diagnostic assessment, cardiovascular implications, and treatment of inherited dyslipidemias. Particular emphasis was placed on studies investigating genetic mechanisms, intracellular lipid trafficking, inflammatory signaling, oxidative imbalance, endoplasmic reticulum stress, microRNA-mediated regulation, intestinal lipid absorption, novel biomarkers, and targeted therapeutic interventions.

The study selection process was conducted independently by two reviewers. Both reviewers screened the titles and abstracts of the retrieved records and subsequently assessed the full texts of potentially relevant publications. Disagreements regarding eligibility or interpretation were resolved through discussion and consensus between the two reviewers.

The literature search retrieved approximately 400 records. After duplicate removal, titles and abstracts were screened for relevance, followed by full-text assessment of potentially eligible studies. Ultimately, 166 publications were included in the final narrative synthesis.

Duplicate records, publications not written in English, articles without accessible full text, and studies that did not directly address the objectives of the review were excluded. Priority was given to recent and methodologically robust studies, landmark publications, and international consensus or guideline documents issued by organizations such as the European Society of Cardiology, European Atherosclerosis Society, American Heart Association, and National Lipid Association.

Data extraction focused on genetic and molecular disease mechanisms, pathways involved in lipoprotein synthesis and clearance, intracellular lipid trafficking, inflammatory and metabolic contributors to atherogenesis, phenotypic variability, diagnostic methodologies, cardiovascular risk stratification, and advances in pharmacological and gene-targeted therapies. The selected evidence was integrated into a narrative synthesis intended to provide a clinically relevant overview of contemporary concepts in familial hypercholesterolemia and familial combined hyperlipidemia.

## 3. Molecular Basis and Genetic Architecture of Primary Hyperlipoproteinemias

**Primary hyperlipoproteinemias** represent a heterogeneous group of inherited disorders of lipoprotein metabolism, characterized by persistent elevations in plasma lipids and lipoproteins, a substantially increased risk of atherosclerotic cardiovascular disease, and, in certain phenotypes, acute pancreatitis. Although traditionally classified according to the Fredrickson phenotypic system, these disorders are now better understood within a molecular and pathophysiological framework that integrates genetics, lipid trafficking, and systemic metabolic regulation (Table 1).

The Fredrickson classification (WHO classification) categorizes hyperlipoproteinemias based on the pattern of lipoprotein elevation identified by electrophoresis or ultracentrifugation.

While this phenotypic system remains clinically useful, it does not capture the underlying genetic complexity. Modern insights reveal that primary hyperlipoproteinemias may result from the following:

- **Monogenic defects** (e.g., LPL, LDLR, APOB, PCSK9 mutations).
- **Polygenic susceptibility** with additive effects of multiple SNPs.
- **Gene–environment interactions**, including diet, obesity, insulin resistance, and epigenetic modulation.

Emerging data further highlight the contribution of intracellular lipid handling, endoplasmic reticulum stress, inflammatory signaling, and dysregulated communication between hepatocytes, adipose tissue, and the intestine. Novel regulators such as ANGPTLs (Angiopoietin-Like Proteins), microRNAs, and pathways governing cholesterol efflux and remnant clearance have refined our understanding of disease heterogeneity.

Thus, the pathogenesis of primary hyperlipoproteinemias bridges classical phenotypic classification with contemporary molecular discoveries, providing a foundation for improved phenotyping, risk stratification, and the development of targeted and precision-based therapeutic strategies.

**Familial hypercholesterolaemia (FH)** is now widely acknowledged as an important public health issue, with a global prevalence estimated at approximately 1 in 300 individuals. Affected individuals face a substantially elevated risk of premature atherosclerotic cardiovascular disease (ASCVD). This heightened risk is primarily driven by markedly increased levels of low-density lipoprotein cholesterol (LDL-C), resulting from pathogenic variants in genes such as LDL receptor (LDLR), apolipoprotein B (APOB), or proprotein convertase subtilisin/kexin type 9 (PCSK9), which disrupt the hepatic receptor-mediated clearance of LDL particles. Beyond the lifelong burden of elevated LDL-C—particularly in cases that remain untreated or insufficiently treated—individuals with FH may also encounter additional cardiovascular risk factors that further amplify ASCVD risk.

Systematic screening for FH is not routinely implemented in most healthcare systems, meaning that clinical presentation often serves as the primary trigger for diagnostic evaluation. In line with this, individuals with obesity were more frequently identified as index cases—that is, the first recognized case of FH within a family. Among children, those with obesity were diagnosed with FH at a slightly younger age—nearly one year earlier on average—compared with their normal-weight peers. In contrast, adults with obesity received an FH diagnosis approximately nine years later than adults of normal weight. These differences may reflect detection biases influenced by health perceptions. Childhood obesity is generally viewed as abnormal and concerning, often prompting medical evaluation and thereby facilitating earlier identification of FH. Conversely, in adult clinical practice, there may be a tendency to attribute hyperlipidemia and/or ASCVD primarily to excess body weight, rather than to investigate an underlying genetic condition such as FH. As a result, obesity in adulthood may inadvertently delay FH recognition. Moreover, obesity can obscure the diagnostic picture by mimicking or overlapping with phenotypically similar conditions, such as mixed dyslipidemia associated with insulin resistance. In some cases, this combined dyslipidemic profile may lead clinicians to diagnose familial combined hyperlipidemia instead of recognizing the coexistence of FH and obesity [3].

Advances in molecular genetics have transformed the diagnostic approach to familial hypercholesterolemia (FH). In many individuals and families with a clinical diagnosis of FH, it is now possible to identify the specific pathogenic variant responsible for the phenotype and establish a definitive genetic diagnosis. Contemporary guidelines strongly recommend confirmatory genetic testing, as it refines cardiovascular risk assessment, informs therapeutic decisions, and facilitates cascade screening of at-risk relatives. The discovery of the LDL receptor (LDLR) gene marked a pivotal milestone in understanding FH, establishing it as the first gene in which mutations were shown to cause the disorder. It is now recognized that autosomal dominant monogenic FH may also result from pathogenic variants in APOB, PCSK9, or APOE—genes encoding proteins directly involved in hepatic clearance of LDL cholesterol. In carriers of these mutations, LDL-C levels are typically elevated to approximately twice the normal concentration. However, considerable variability exists, as the clinical severity depends on the specific genetic defect. In general, complete loss-of-function variants are associated with markedly higher LDL-C levels and earlier onset of coronary heart disease (CHD), whereas variants retaining partial protein activity tend to produce a milder phenotype. Although most cases of FH follow an autosomal dominant inheritance pattern, rare forms of recessive hypercholesterolemia have also been described. These include pathogenic variants in LDLRAP1, which impairs LDL receptor internalization and recycling, as well as mutations in ABCG5 or ABCG8, which cause sitosterolemia through increased intestinal absorption of plant sterols. Additional rare defects affecting hepatic lipid metabolism, such as lysosomal acid lipase deficiency (LALD), can also present with hypercholesterolemia [4,5]. From a genetic perspective, FH is best considered a co-dominant condition: individuals carrying one pathogenic variant are classified as having heterozygous FH (HeFH), whereas those with two pathogenic variants—either homozygous or compound heterozygous—are diagnosed with homozygous FH (HoFH), a substantially more severe clinical entity [6].

### 3.1. Genes in Which Pathogenic Variants Cause Monogenic Familial Hypercholesterolemia

#### 3.1.1. LDL Receptor (LDLR)

Because familial hypercholesterolemia (FH) is fundamentally a disorder of LDL cholesterol (LDL-C) clearance, understanding the physiology of LDL metabolism is essential. LDL particles consist primarily of a single apolipoprotein B-100 (apoB100) molecule that surrounds a lipid core rich in cholesteryl esters and triglycerides, along with smaller amounts of additional lipid components. Under normal conditions, circulating LDL particles bind to LDL receptors expressed on the hepatocyte surface through the apoB100 ligand. This interaction triggers internalization of the LDL–receptor complex into endosomes within the hepatocyte. Inside the cell, LDL particles are degraded into their lipid and protein constituents. The LDL receptor is then either recycled back to the plasma membrane for reuse or targeted to lysosomal degradation. Disruption at any stage of this pathway—binding, internalization, processing, or receptor recycling—can impair LDL clearance and lead to elevated plasma LDL-C levels, the hallmark of FH. The LDLR gene was the first gene identified in which mutations were shown to cause FH. Located on the short arm of chromosome 19, it spans approximately 45 kilobases and contains 18 exons encoding the LDL receptor protein. The mature receptor comprises five functional domains responsible for ligand binding, receptor clustering, internalization, and recycling. Pathogenic variants that reduce or abolish LDL receptor function result in diminished hepatic uptake of LDL-C and persistent hypercholesterolemia. Mutations in LDLR are distributed throughout the entire gene. To date, more than 2300 distinct variants have been reported. Most are exonic substitutions, small insertions or deletions (<100 base pairs), or larger genomic rearrangements (>100 base pairs). Certain variants have higher prevalence in specific populations due to founder effects associated with historical migration and population expansion. Notable examples include well-characterized mutations in Finland, South Africa, and French Canadian populations. Overall, LDLR mutations remain the most common genetic cause of monogenic FH, with phenotypic severity varying according to whether the mutation results in complete loss of receptor function or partial residual activity [7,8,9].

#### 3.1.2. Apolipoprotein B-100

Apolipoprotein B-100 (apoB-100) is the principal structural protein of LDL particles and serves as the ligand for the LDL receptor, enabling hepatic clearance of LDL cholesterol. The APOB gene, located on chromosome 2p, spans over 43 kb and contains 29 exons encoding a large 4563-amino-acid protein. While truncating mutations in APOB lead to hypobetalipoproteinemia, hypercholesterolemia results from specific missense variants that impair the binding of LDL particles to the LDL receptor. This condition is known as familial defective ApoB-100 (FDB). Clinically, FDB resembles LDLR-related FH but is typically milder on average. The most common pathogenic variant is p.(Arg3527Gln), which markedly reduces LDL receptor affinity and LDL clearance. In non-Finnish European populations, its frequency (~0.00049 in gnomAD v4.1.0) makes it the most frequent single genetic cause of the FH phenotype in individuals of European ancestry. Haplotype studies suggest a founder origin in Western Europe approximately 6000–7000 years ago, with subsequent geographic spread across much of Europe but near absence in Finland and Greece. Another mutation at the same residue, p.(Arg3527Trp), has been identified primarily in individuals of South and East Asian ancestry (frequency ~0.00042), where it likely represents the most common monogenic cause of the FH phenotype in those populations. Although more than 350 APOB variants have been reported, only a small proportion (~10%) are considered pathogenic. Interpretation remains challenging due to the large size and complex structure of apoB-100. Recent structural insights from cryo-electron microscopy have improved understanding of LDL receptor–apoB interactions, potentially aiding future classification of APOB variants. Overall, APOB mutations represent an important but less common cause of monogenic FH compared with LDLR mutations, with phenotype severity generally influenced by the degree of impairment in LDL receptor binding [10].

#### 3.1.3. PCSK9

The PCSK9 gene (proprotein convertase subtilisin/kexin type 9), located on chromosome 1p and comprising 12 exons, encodes a protein that regulates LDL receptor degradation. PCSK9 is synthesized as an inactive precursor in the endoplasmic reticulum and then secreted—primarily by the liver. When circulating PCSK9 binds to the LDL receptor, it directs the receptor toward lysosomal degradation rather than recycling to the hepatocyte surface, thereby reducing LDL receptor availability and increasing plasma LDL-C levels. PCSK9 was identified as an FH-causing gene through linkage analysis in families with a clinical FH phenotype but no detectable LDLR or APOB mutations. Pathogenic variants associated with FH are gain-of-function mutations, which enhance LDL receptor degradation and reduce receptor density at the cell surface. More than 20 such variants have been described globally. In contrast, loss-of-function variants in PCSK9 lead to reduced LDL receptor degradation, enhanced LDL clearance, and lower LDL-C levels. The most common example, p.(Arg46Leu), occurs in about 3% of individuals of European ancestry and is associated with lifelong lower LDL-C and an approximately 28% reduction in coronary heart disease risk. Rare structural variants, such as complete duplication of the PCSK9 gene, have also been reported and can result in markedly elevated PCSK9 levels and resistance to statin therapy, producing an FH phenotype. Interpreting PCSK9 variants is more challenging than for LDLR or APOB. In silico tools may predict functional impact but cannot reliably distinguish gain-of-function (FH-causing) from loss-of-function (protective) variants. Among reported PCSK9 variants, only a minority are classified as pathogenic, while a substantial proportion remain variants of uncertain significance (VUS) [11,12].

#### 3.1.4. Apolipoprotein E

Apolipoprotein E (ApoE) is a 34 kDa multifunctional protein primarily synthesized in the liver and present on triglyceride-rich lipoproteins, including chylomicrons, VLDL, and their remnants. The APOE gene, located on chromosome 19 (distinct from the LDLR locus), spans approximately 3.6 kb and contains four exons. ApoE facilitates high-affinity binding of lipoprotein particles to the LDL receptor, thereby promoting their hepatic clearance. APOE is polymorphic, with three common isoforms—E2, E3, and E4—determined by two nonsynonymous SNPs in exon 4. These isoforms differ at amino acid positions 112 and 158 and influence lipid metabolism in an isoform-dependent manner. In populations of European ancestry, the E3 isoform (Cys112, Arg158) is most prevalent (~79%), followed by E4 (~14%, Arg112, Arg158) and E2 (~7%, Cys112, Cys158). The six common genotypes, in decreasing frequency, are ε3/ε3, ε3/ε4, ε3/ε2, ε2/ε4, ε4/ε4, and ε2/ε2. Although these genotypes significantly modulate plasma lipid levels, they are not considered causative of classical FH. However, a rare APOE variant—p.(Leu167del)—has been identified as a monogenic cause of autosomal dominant hypercholesterolemia. This in-frame three-base pair deletion (c.500_502delTCC) was discovered through genome-wide linkage analysis and whole-exome sequencing in a family exhibiting an FH phenotype without mutations in LDLR, APOB, or PCSK9. Functional modeling suggested that deletion of leucine at position 167 disrupts an alpha-helix within the LDL receptor-binding domain, impairing ApoE function. Subsequent in vivo studies demonstrated that this variant leads to reduced LDL receptor expression on hepatocytes and diminished clearance of ApoE-containing lipoproteins, thereby producing an FH-like phenotype. Recent population data indicate that p.(Leu167del) is rare in the general population (minor allele frequency ~8 × 10^−5^), but significantly enriched among individuals with clinically diagnosed FH, supporting its pathogenic role. In summary, while common APOE isoforms modulate lipid levels without causing FH, rare structural variants such as p.(Leu167del) can produce a true autosomal dominant hypercholesterolemia phenotype [13,14].

### 3.2. The Role of the Lipoprotein a Gene in the FH Phenotype

**Figure 2:**
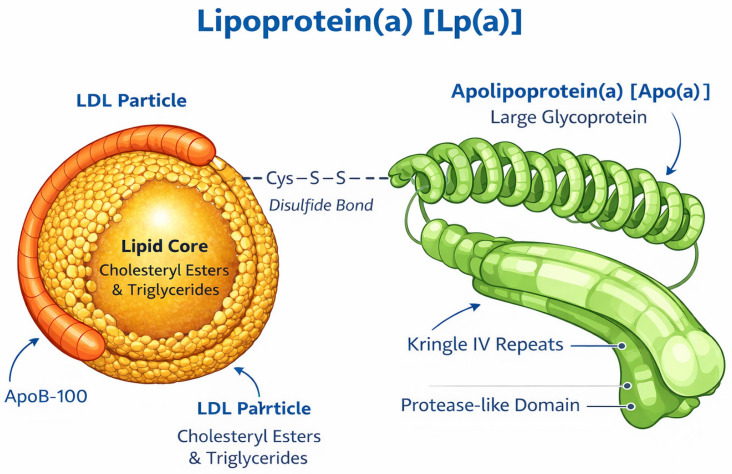
Lipoprotein A.

Lipoprotein(a) [Lp(a)] is a lipoprotein particle composed of an LDL-like core covalently bound to apolipoprotein(a) [apo(a)] via a disulfide bridge linking apo(a) to apolipoprotein B-100. Apo(a) shares structural homology with plasminogen, a key protein in fibrinolysis, and is encoded by the LPA gene located on chromosome 6q26. Unlike LDL-C—whose plasma concentration is largely determined by hepatic receptor-mediated clearance—Lp(a) levels are primarily governed by hepatic production rates. Circulating Lp(a) concentrations are minimally influenced by diet or lifestyle and are instead strongly genetically determined. Genetic variation at the LPA locus includes coding variants, regulatory polymorphisms affecting expression and splicing, and, most importantly, copy number variation in the kringle IV type 2 (KIV-2) repeat region. The number of KIV-2 repeats inversely correlates with circulating Lp(a) levels: alleles with fewer repeats produce smaller apo(a) isoforms and are associated with higher plasma Lp(a) concentrations, whereas longer isoforms correspond to lower levels. An individual’s total Lp(a) concentration reflects the combined contribution of both parental alleles. In European populations, variation in KIV-2 copy number accounts for approximately 60–70% of interindividual variability in Lp(a) levels. Additionally, a genetic risk score based on multiple SNPs within the LPA locus has been shown to strongly predict circulating Lp(a) concentrations. Elevated Lp(a) is a well-established independent risk factor for coronary heart disease (CHD), with strong support from Mendelian randomization studies demonstrating a causal association between LPA variants and cardiovascular risk. The combination of high LDL-C and elevated Lp(a) confers particularly marked risk amplification. Lp(a) may contribute to the marked heterogeneity in cardiovascular risk observed among patients with familial hypercholesterolemia, highlighting its potential value in individualized risk stratification (Figure 2) [15].

#### Clinical Challenges and Emerging Therapeutic Perspectives

Despite the growing recognition of Lp(a) as an independent cardiovascular risk factor, several challenges continue to limit its routine clinical implementation. Measurement of Lp(a) remains complicated by the marked heterogeneity in apo(a) isoform size, which may influence assay performance despite ongoing efforts toward assay standardization. Contemporary guidelines recommend reporting Lp(a) concentrations preferentially in nmol/L rather than mg/dL, although both units remain in clinical use and are not directly interchangeable because of interindividual differences in apo(a) isoform size. In addition, the optimal threshold defining elevated Lp(a) remains a matter of debate, with current recommendations generally considering concentrations ≥50 mg/dL (approximately ≥125 nmol/L) as clinically significant, while recognizing that cardiovascular risk increases continuously across the Lp(a) distribution rather than above a single universal cut-off.

Current lipid-lowering therapies have limited effects on Lp(a) concentrations. Statins generally have little effect or may modestly increase Lp(a), whereas PCSK9 inhibitors reduce circulating levels by approximately 20–30%. Consequently, considerable interest has focused on novel Lp(a)-targeted therapies. RNA-based approaches, including antisense oligonucleotides such as pelacarsen and small interfering RNAs (siRNAs) including olpasiran, zerlasiran, and lepodisiran, have demonstrated substantial reductions in circulating Lp(a) concentrations in phase 2 clinical trials. Whether these profound reductions translate into improved cardiovascular outcomes is currently being investigated in large randomized outcome studies, including the Lp(a) HORIZON and OCEAN(a) trials. These ongoing studies are expected to clarify whether selective Lp(a) lowering provides incremental cardiovascular benefit beyond intensive LDL-C reduction and may establish Lp(a) as a therapeutic target in precision cardiovascular medicine.

### 3.3. STAP1

In 2014, STAP1 (signal transducing adaptor family member 1) was proposed as a novel candidate gene for familial hypercholesterolemia (FH) following linkage analysis in a large Dutch family exhibiting a clinical FH phenotype without detectable variants in LDLR, APOB, or PCSK9. Three genomic regions achieved significant linkage (LOD score of 3.0), and sequencing within these regions identified a potentially pathogenic variant in STAP1, located on chromosome 4p15.1–q13.3. Additional variants in STAP1 were subsequently reported in other individuals with hypercholesterolemia, further supporting its candidacy at the time. STAP1—also known as BRDG1 (BCR downstream signaling protein 1)—encodes an adaptor protein containing a pleckstrin homology (PH) domain, a Src homology 2 (SH2) domain, and multiple tyrosine phosphorylation sites. Despite these signaling-related domains, there was no clear biological link between STAP1 and lipid metabolism or LDL receptor–mediated clearance, raising early mechanistic doubts. Subsequent investigations, including functional studies and rigorous co-segregation analyses in affected families, failed to demonstrate a causal relationship between STAP1 variants and hypercholesterolemia. These findings ultimately refuted its role as an FH gene. The STAP1 case highlights the importance of stringent validation—including functional evidence and robust genetic segregation data—before establishing new genes as causative in monogenic disorders such as FH [16].

### 3.4. The Polygenic Basis of the FH Phenotype

Among individuals with a strong clinical suspicion of familial hypercholesterolemia (e.g., Simon Broome “definite FH” or Dutch Lipid Clinic Network score >8), a monogenic cause can be identified in approximately 40–80% of cases. However, in patients with a lower clinical probability, the diagnostic yield falls to 20–30%. In those who meet clinical criteria but lack a detectable pathogenic variant in LDLR, APOB, PCSK9, or other established genes, a polygenic origin should be considered. A polygenic FH phenotype arises from the cumulative inheritance of multiple common LDL-C–raising single nucleotide polymorphisms (SNPs), each contributing modestly to lipid levels. Genome-wide association studies (GWAS) have identified numerous loci associated with plasma lipid concentrations. Building on these findings, LDL-C genetic risk scores (GRS) have been developed using weighted combinations of LDL-C-raising alleles. Studies in UK and international cohorts demonstrate that in more than 80% of clinically diagnosed FH patients without a monogenic mutation, elevated LDL-C is likely driven by a polygenic predisposition [17,18].

Interestingly, even individuals with a confirmed monogenic mutation tend to have intermediate LDL-C genetic risk scores compared with mutation-negative FH patients and healthy controls. This suggests that polygenic background may further modulate LDL-C levels and contribute to phenotype severity even in monogenic FH. Polygenic burden also influences coronary heart disease (CHD) risk. Among individuals heterozygous for an FH-causing mutation, those with a higher LDL-C GRS have a greater risk of CHD than mutation carriers with a lower GRS. Thus, genetic risk scores provide additional stratification beyond monogenic status alone. From a clinical perspective, LDL-C genetic risk scoring has practical implications. Studies comparing individuals with polygenic hypercholesterolemia to those with monogenic FH have shown that, despite similar LDL-C levels at the time of evaluation, monogenic FH carriers exhibit greater subclinical atherosclerosis and coronary artery calcification. Large biobank analyses confirm that while a high LDL-C GRS is associated with elevated CHD risk, this risk remains substantially lower than that observed in individuals with heterozygous monogenic FH. The most plausible explanation lies in cumulative LDL-C exposure. Monogenic FH carriers are exposed to markedly elevated LDL-C from birth, resulting in a higher lifelong LDL-C burden compared with individuals whose LDL-C levels increase gradually due to polygenic influences. Consequently, while individuals with polygenic hypercholesterolemia are at increased cardiovascular risk and require lipid-lowering therapy, the substantially higher risk associated with monogenic FH justifies the more aggressive treatment strategies recommended in current guidelines [19] (Table 2).

**Summary of Key Differences**

- **Monogenic FH** results from a single pathogenic variant with large effect size and causes markedly elevated LDL-C from birth.
- **Polygenic hypercholesterolemia** results from accumulation of common LDL-C-raising variants and typically produces a milder but still clinically relevant phenotype.
- **LPA variation** independently modifies cardiovascular risk and may amplify risk in FH.
- Cardiovascular risk is strongly influenced by cumulative LDL-C exposure, explaining why monogenic FH carries substantially higher risk than polygenic forms even at similar LDL-C levels measured later in life.

Current guidelines stress the importance of initiating cholesterol-lowering therapy early in life to prevent coronary artery disease, particularly in patients with homozygous (HoFH) and severe heterozygous familial hypercholesterolemia (HeFH), as cardiovascular risk correlates strongly with cumulative LDL-C exposure. Early treatment—ideally beginning in childhood—yields the most favorable long-term outcomes and is cost-effective. Encouragingly, several LDL-C-lowering therapies are now approved for use in children and adolescents, with additional agents under clinical investigation. Advances in achieving lower LDL-C levels, combined with improved vascular imaging for detecting subclinical atherosclerosis, offer valuable opportunities to better define the natural history of FH and refine therapeutic strategies. Although current guidelines provide clear LDL-C-based thresholds for initiating therapy in children and adolescents, substantial uncertainty remains regarding optimal lifelong management of familial hypercholesterolemia (FH). Key unresolved issues include whether treatment intensity should be guided solely by LDL-C levels or refined through broader cardiovascular risk stratification. The potential role of vascular imaging in monitoring therapeutic response and subclinical atherosclerosis progression also warrants further clarification.

Special clinical scenarios pose additional challenges. During pregnancy, most lipid-lowering therapies are contraindicated and must be discontinued—raising questions about the duration of treatment interruption, particularly in women who breastfeed. Similarly, the safety and feasibility of temporary “drug holidays” remain uncertain. With the prospect of newborn screening enabling diagnosis at birth, important questions arise regarding preventive strategies during the first decade of life. Personalized care must also account for individual circumstances, family experience with FH, and psychosocial factors. Management of older patients with FH who have no prior cardiovascular events and no detectable atherosclerosis presents another clinical dilemma. Finally, disparities in healthcare systems worldwide significantly influence early diagnosis, access to therapy, and the timely initiation of care, underscoring the need for adaptable, resource-sensitive strategies in FH management. (FH) can be diagnosed either by identifying a pathogenic variant in an FH-associated gene or by applying validated clinical scoring systems. Commonly used phenotypic tools include the Dutch Lipid Clinic Network (DLCN) criteria and the Simon–Broome criteria. Importantly, there is not always complete concordance between genotype and phenotype: some individuals carrying a pathogenic mutation may not meet clinical diagnostic thresholds, whereas others who fulfill phenotypic criteria may have negative genetic testing. Genetic testing, while considered the diagnostic gold standard, serves additional purposes beyond confirmation. It facilitates cascade screening among relatives and helps distinguish heterozygous FH (HeFH) from homozygous FH (HoFH), particularly in cases of severe hypercholesterolemia. However, in many parts of the world, access to genetic testing remains limited due to cost or availability. Consequently, outside countries with systematic cholesterol screening programs, most children are diagnosed through cascade testing from an index case or based on a strong family history of FH, premature coronary artery disease, or markedly elevated LDL-C levels [3,20] (Table 3).

In clinical practice, both systems are widely used. The DLCN criteria provide a more refined, graded probability of diagnosis, while the Simon–Broome criteria offer a straightforward categorical framework. Selection often depends on regional practice patterns, availability of genetic testing, and clinical context.

### 3.5. Homozygous Familial Hypercholesterolemia (HoFH): Urgency, Multidisciplinary Care, and Advanced Monitoring

Although homozygous familial hypercholesterolemia (HoFH) is rare, early diagnosis and immediate intensive treatment are critical. Without timely intervention, HoFH can lead to rapidly progressive atherosclerosis, juvenile-onset cardiovascular disease, and premature mortality. In untreated individuals, fatal or nonfatal myocardial infarction and severe aortic stenosis may occur within the first two decades of life.

**Figure 3:**
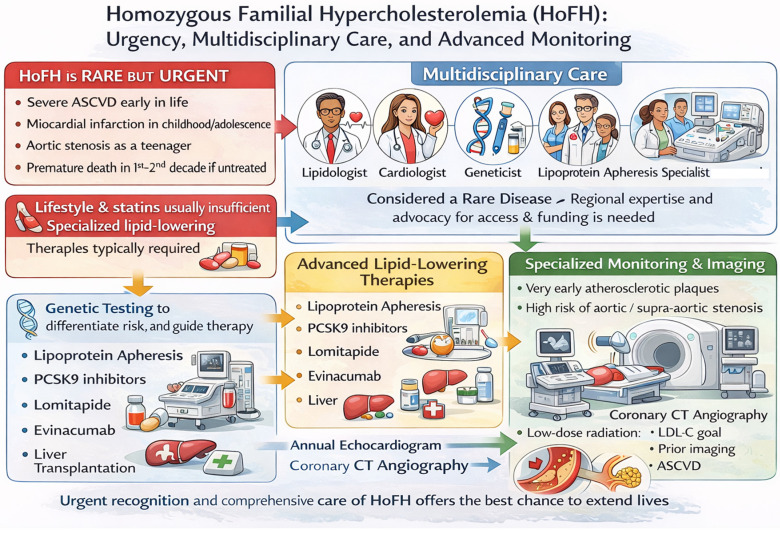
Homozygous Familial Hypercholesterolemia (HoFH): Early Detection, Multidisciplinary Management, and Advanced Cardiovascular Monitoring.

Conventional measures—such as lifestyle modification and high-intensity statin therapy—are generally insufficient to achieve adequate LDL-C reduction in HoFH. Most patients require advanced lipid-lowering strategies, and in many countries, recognition of HoFH as a rare disease eligible for dedicated government funding is essential to ensure access to appropriate care. Optimal management should take place in specialized centers with multidisciplinary teams experienced in lipidology, cardiology, genetics, and lipoprotein apheresis. Comprehensive care also depends on allied health professionals—including nurses, dietitians, pharmacists, and counselors—who contribute significantly to adherence and long-term outcomes. Treatment should begin immediately at diagnosis and often includes specialized therapies such as lipoprotein apheresis; in rare cases, liver transplantation may be considered. Given the very early development of atherosclerotic plaques and the risk of aortic or supra-aortic stenosis, systematic imaging surveillance is indispensable. Advances in low-dose computed tomography (CT) technology have improved the feasibility of coronary CT angiography in selected pediatric patients. However, routine coronary CT angiography is not recommended for the evaluation of all children with familial hypercholesterolemia. Its use should be individualized and reserved for selected high-risk cases—such as patients with homozygous familial hypercholesterolemia or those with suspected premature cardiovascular involvement—following multidisciplinary assessment. Decisions regarding the timing and frequency of imaging should be guided by the patient’s clinical phenotype, cardiovascular risk profile, treatment response, and potential impact on management. Echocardiography may be considered in selected patients, particularly those with homozygous familial hypercholesterolemia or clinically suspected valvular or supravalvular aortic involvement. The frequency of follow-up should be individualized according to the patient’s clinical status, baseline imaging findings, and cardiovascular risk, and can also be performed routinely on an annual basis [21,22,23]. Genetic testing complements the diagnostic evaluation by confirming the molecular diagnosis, facilitating cascade screening, and contributing to cardiovascular risk assessment. Certain pathogenic variants, particularly LDLR null variants, have been associated with higher LDL-C concentrations and a greater lifetime risk of ASCVD compared with receptor-defective variants. However, prognosis is influenced not only by genotype but also by cumulative LDL-C exposure, additional cardiovascular risk factors, and treatment intensity. Consequently, genetic findings should be interpreted within the broader clinical context rather than considered independent prognostic determinants (Figure 3).

In children and adolescents with FH, statins remain the cornerstone of therapy and are the only class with extensive long-term safety and efficacy data in this population. Their mechanism—HMG-CoA reductase inhibition leading to upregulation of LDL receptors—aligns well with the pathophysiology of FH and allows for meaningful LDL-C reductions (20–50%). Ezetimibe is commonly used as adjunctive therapy when LDL-C targets are not achieved with statins alone, offering additional modest LDL-C lowering through inhibition of intestinal cholesterol absorption.

More recently, PCSK9 monoclonal antibodies (alirocumab and evolocumab) have expanded therapeutic options in pediatric patients, particularly in those with inadequate response to statins or statin intolerance. These agents enhance LDL receptor recycling and provide substantial additional LDL-C reduction (≈30–40%). For severe cases—especially homozygous FH (HoFH)—evinacumab, an ANGPTL3 inhibitor, offers a receptor-independent mechanism of LDL-C reduction and has become a critical therapeutic option.

In contrast, several newer lipid-lowering agents—such as inclisiran (siRNA targeting PCSK9) and bempedoic acid (ATP-citrate lyase inhibitor)—are currently approved only for adults and remain under evaluation in pediatric populations. While these therapies offer substantial LDL-C reductions in adults, long-term safety, growth implications, and developmental considerations must be clarified before routine pediatric use.

Similarly, lomitapide, an inhibitor of microsomal triglyceride transfer protein (MTP), is primarily reserved for HoFH and remains restricted due to tolerability concerns, hepatic effects, and dietary constraints. Its pediatric use is still limited despite encouraging LDL-C reductions in small HoFH cohorts.

An important distinction between pediatric and adult therapy lies not only in drug approval status but also in treatment philosophy. In children, therapy prioritizes long-term safety, preservation of normal growth and development, and early prevention of cumulative LDL-C exposure. In adults, broader therapeutic combinations—including newer oral agents—may be used more aggressively to achieve intensive LDL-C targets, particularly in secondary prevention.

Overall, pediatric lipid management emphasizes early initiation, stepwise intensification, and careful safety monitoring, whereas adult therapy allows for a wider pharmacologic armamentarium with more extensive outcome data. Continued clinical trials will help bridge this gap and expand evidence-based options for children with FH [24] (Table 4).

### 3.6. Current Treatment Guidance in Familial Hypercholesterolemia

LDL-C treatment goals in familial hypercholesterolemia (FH) should be individualized according to age and overall cardiovascular risk. In children, the recommended treatment target is generally < 135 mg/dL (3.5 mmol/L). In adults, current ESC/EAS guidelines recommend both a ≥50% reduction in LDL-C from baseline and achievement of risk-specific LDL-C goals. For adults at high cardiovascular risk, the target LDL-C is <70 mg/dL (<1.8 mmol/L), whereas for those at very high cardiovascular risk, including patients with established ASCVD or another major risk factor, the recommended target is <55 mg/dL (<1.4 mmol/L). In patients experiencing recurrent ASCVD events within two years despite maximally tolerated lipid-lowering therapy, an LDL-C goal <40 mg/dL (<1.0 mmol/L) may be considered. These thresholds are largely derived from expert consensus and indirect evidence rather than randomized trials specifically conducted in FH populations. Because many adults require combination therapy (often two or more agents), careful monitoring of efficacy and safety is essential. Management should also address comorbid conditions—including obesity, diabetes, hypertension, and psychological factors—according to standard clinical guidelines. Measurement of lipoprotein(a) is recommended, and if elevated, further intensification of LDL-C lowering and global cardiovascular risk management is advised. In patients with established atherosclerotic cardiovascular disease, care should follow secondary prevention protocols. In pediatric FH, the safety and effectiveness of treatment have been demonstrated in trials evaluating statins, ezetimibe, colesevelam, and PCSK9 inhibitors. High-intensity statins can reduce LDL-C by approximately 50%, while adjunctive therapies achieve additional reductions (colesevelam ≈12%, ezetimibe ≈27%, PCSK9 inhibitors ≈44%). Importantly, statin therapy initiated in childhood has been associated with regression or stabilization of carotid intima-media thickness (CIMT), a surrogate marker of early atherosclerosis. Extension studies indicate that PCSK9 inhibitors remain well tolerated for up to two years in pediatric populations, and long-term observational data confirm a favorable safety profile for statins, without evidence of increased muscle toxicity, liver dysfunction, or incident diabetes. A landmark longitudinal study of individuals with heterozygous FH treated with statins from childhood and followed into adulthood demonstrated markedly lower rates of CAD and mortality compared with their affected but untreated parents. Despite achieved LDL-C levels that were higher than current pediatric targets, treated individuals had dramatically reduced cardiovascular events and CIMT values comparable to unaffected siblings, supporting both the long-term efficacy and safety of early intervention. However, current guidelines do not fully account for cumulative lifetime LDL-C exposure—the so-called “cholesterol burden.” Even patients treated from youth may exhibit residual subclinical atherosclerosis in midlife, likely reflecting prolonged exposure to LDL-C levels that, although reduced, remain above those of the general population. Because present recommendations are based primarily on LDL-C values measured at clinical encounters rather than cumulative exposure, individuals who begin therapy later in life may require more aggressive lipid lowering to mitigate the impact of longstanding hypercholesterolemia [24,25].

Most lipid-lowering agents, including statins, ezetimibe, and PCSK9 inhibitors, are generally discontinued before conception and during pregnancy because of limited safety data. However, management should be individualized according to maternal cardiovascular risk, particularly in women with homozygous FH or established ASCVD. Lipoprotein apheresis remains an option in selected high-risk patients.

**Familial combined hyperlipidemia (FCHL)** was initially reported in 1973 by Goldstein and colleagues in Seattle [26]. Rose et al. [27] and Nikkilä and Aro [28] independently described a comparable lipid disorder, reinforcing recognition of this clinical entity. Goldstein and coworkers proposed that FCHL was the most prevalent autosomal dominant disorder of lipid metabolism and a major contributor to premature atherosclerosis—an assertion that was confirmed by epidemiological and genetic studies in the decades that followed. Early research aimed to identify a single causative gene responsible for FCHL; however, these efforts did not yield definitive results. Current understanding supports a polygenic model, whereby multiple genetic variants collectively contribute to the expression of the disorder. In addition, there is growing indirect evidence that rare variants with incomplete penetrance, together with the additive effects of common susceptibility alleles, may underline the broader phenotype referred to as combined or multiple-type hyperlipidemia. A hallmark of FCHL is its marked intrafamilial variability: distinct lipid phenotypes—such as isolated hypercholesterolemia, hypertriglyceridemia, or mixed dyslipidemia—may coexist within the same pedigree. Individuals affected by FCHL carry a substantially increased risk of cardiovascular complications and are also predisposed to metabolic comorbidities, including type 2 diabetes mellitus and steatotic liver disease. Several metabolic abnormalities are consistently observed in individuals with familial combined hyperlipidemia (FCHL). One of the most extensively studied and earliest recognized features is hepatic overproduction of very-low-density lipoprotein (VLDL). Closely linked to this disturbance is insulin resistance, which is considered a key metabolic characteristic of the condition. As a likely consequence of excessive VLDL production, patients often exhibit impaired clearance of chylomicron remnants and elevated levels of atherogenic lipoproteins retained along the vascular endothelium, both contributing to increased cardiovascular risk. A proposed underlying mechanism for hepatic VLDL overproduction involves abnormal plasma free fatty acid (FFA) metabolism. Specifically, inefficient peripheral uptake of FFAs may result in increased hepatic FFA delivery, thereby stimulating VLDL synthesis. Limited in vivo evidence supports this hypothesis, including a small study demonstrating exaggerated postprandial ketone body production in individuals with FCHL, suggesting enhanced hepatic fatty acid flux. This concept is partly grounded in earlier work indicating impaired function of acylation-stimulating protein (ASP) in individuals with elevated apolipoprotein B (hyperapoB). ASP was later identified as C3adesArg, a derivative of complement component C3. Subsequent studies have linked complement system activity—particularly C3—with triglyceride-rich lipoprotein metabolism, postprandial lipid handling, and the FCHL phenotype. In one family, a mutation in the C5L2 gene, encoding the ASP receptor, was associated with phenotypic expression of FCHL, further implicating complement pathways. Despite these associations, and increasing insight into the interaction between complement activation and lipoprotein metabolism, the precise pathogenetic role of the complement system in FCHL remains incompletely understood [29] (Table 5).

### 3.7. FCHL Is Genetically Heterogeneous and Polygenic

It is highly plausible that different genetic variants can produce the same FCHL phenotype, meaning that each affected family may carry a distinct genetic background. This genetic heterogeneity helps explain why genome-wide association studies (GWASs) have failed to identify a single causative gene. Over time, research has clearly moved away from a monogenic model toward recognition of FCHL as a polygenic disorder, with an important contribution from triglyceride-raising single nucleotide polymorphisms (SNPs). Following the initial description of FCHL in 1973, the disorder appeared to follow an autosomal dominant inheritance pattern. This led to decades of linkage analyses aimed at identifying a major gene effect. Several genomic regions were identified as potentially relevant, but none proved universally causative. One of the earliest findings implicated the APOA1-C3-A4 gene cluster, later expanded to include APOA5, a gene strongly associated with triglyceride levels. Variants in this region were overrepresented in some FCHL families, particularly in Northern European populations. However, these associations were not consistently reproducible across all cohorts. Another promising region identified through linkage studies was chromosome 1q21-23, with USF1 (Upstream Stimulating Factor 1) emerging as a candidate gene involved in lipid, glucose, and adipose tissue metabolism. Although these loci appeared important in certain pedigrees, they could not fully explain FCHL as a single-gene disorder [30,31].

### 3.8. Shift Toward a Polygenic Risk Model

The inability to identify a universal causal gene, combined with growing evidence from GWAS demonstrating the impact of common variants in complex diseases, shifted the understanding of FCHL toward a polygenic framework. Subsequent studies using polygenic risk scores (PRS) confirmed this concept. PRS analyses consistently showed that approximately 25% of individuals with FCHL exhibit an excess burden of triglyceride-raising SNPs, while LDL-C–raising variants appear less prominently represented. Many of these SNPs are located in novel or regulatory genomic regions, suggesting that additional functional mechanisms remain to be clarified. Although FCHL is now considered polygenic, decades of family-based studies have identified numerous genes that may modulate its phenotype. These genes primarily affect the following:

- Triglyceride metabolism (e.g., LPL, APOC3, APOA5, LIPC, CETP, GPIHBP1).
- Hepatic VLDL production and fat accumulation (e.g., GCKR).
- Adipose tissue function (e.g., USF1).
- LDL metabolism (e.g., LDLR, PCSK9, SREBP-2).

However, most of these variants are not exclusive to FCHL and are also found in individuals with isolated hypertriglyceridemia. An interesting candidate gene is ANGPTL3, whose loss-of-function mutations cause familial combined hypolipidemia—the metabolic opposite of FCHL. Nonetheless, a direct causal role of ANGPTL3 in FCHL has not been firmly established [32,33].

The Maastricht group investigated the relationship between serum triglycerides and LDL cholesterol to clarify the heterogeneity of multiple-type hyperlipidemia. Based on the observation that triglyceride-rich VLDL1 particles—particularly when serum triglycerides exceed 1.5 mmol/L—exchange triglycerides for cholesteryl esters from LDL particles, they proposed a parabolic relationship between triglycerides and LDL-C. This pattern was confirmed in two FCHL cohorts, although it was not specific to FCHL, as it was also observed in the general population and in individuals with type 2 diabetes. In FCHL, genetic predisposition toward hypertriglyceridemia promotes progression along this parabolic curve, while a genetic tendency toward elevated cholesterol shifts the curve upward, influencing the threshold for hypercholesterolemia. Consequently, individuals with FCHL may present variably with hypercholesterolemia, hypertriglyceridemia, or combined hyperlipidemia, depending on their current triglyceride levels. Longitudinal studies have shown that fluctuations in the hypertriglyceridemic phenotype are closely linked to body mass index, insulin resistance, and intrahepatic lipid content. Changes in liver fat—potentially even after a single high-fat meal—can alter insulin-mediated VLDL production and shift an individual’s position along the parabolic curve. This metabolic variability contributes to the diagnostic complexity of FCHL in clinical practice. FCHL was originally described in families with premature myocardial infarction, suggesting that the elevated cardiovascular risk observed in these pedigrees may not be attributable to dyslipidemia alone. Additional genetic and environmental factors likely contribute. Therefore, a positive family history of premature cardiovascular disease should be considered when defining treatment targets in patients with dyslipidemia. Importantly, LDL-C may underestimate cardiovascular risk in individuals with hypertriglyceridemia because of the presence of atherogenic remnant particles and small dense LDL. For this reason, non-HDL cholesterol—or preferably apolipoprotein B—should be used as secondary treatment targets. Moreover, the increased risk of type 2 diabetes, particularly in patients with hypertriglyceridemia, must be acknowledged. Assessing intrahepatic fat (via ultrasound or MRI) may improve risk stratification, as patients with steatotic liver disease are likely to benefit substantially from intensive lifestyle interventions. Rather than a single-gene disorder, FCHL represents a cluster of susceptibility genes—relatively homogeneous within families but heterogeneous across pedigrees—that manifest under environmental influence. This concept aligns with the polygenic nature of other metabolic conditions such as obesity, MASLD (Metabolic dysfunction–Associated Steatotic Liver Disease), and type 2 diabetes. Emerging data-driven subclassifications in these disorders suggest that a similar approach may refine the understanding of polygenic lipid disorders. Until such precision strategies are implemented, individuals with an FCHL phenotype should be managed aggressively to reduce cardiometabolic risk [34,35,36].

## 4. Diagnostic Strategies in Familial Hypercholesterolemia and Familial Combined Hyperlipidemia

Early and accurate diagnosis of inherited dyslipidemias is essential to enable timely initiation of lipid-lowering therapy, reduce lifelong exposure to atherogenic lipoproteins, and facilitate cascade screening of affected relatives. The diagnostic approach should integrate clinical assessment, laboratory evaluation, genetic testing, and cardiovascular risk stratification rather than relying on any single diagnostic modality.

### 4.1. Initial Clinical Evaluation and Exclusion of Secondary Dyslipidemias

The diagnostic evaluation begins with a detailed personal and family history, including premature atherosclerotic cardiovascular disease (ASCVD), tendon xanthomas, corneal arcus, and known familial lipid disorders. Before establishing the diagnosis of a primary hyperlipoproteinemia, secondary causes of dyslipidemia should be systematically excluded. Common secondary causes include hypothyroidism, diabetes mellitus, nephrotic syndrome, chronic kidney disease, cholestatic liver disease, obesity and metabolic syndrome, excessive alcohol consumption, pregnancy, and medications such as glucocorticoids, thiazide diuretics, retinoids, antiretroviral agents, and immunosuppressive drugs. Appropriate laboratory investigations, including thyroid function tests, renal and liver function tests, fasting glucose or glycated hemoglobin, and urinary protein assessment, are recommended whenever clinically indicated.

### 4.2. Interpretation of the Lipid Profile

Whenever possible, lipid concentrations should be interpreted before initiation of lipid-lowering therapy. In patients already receiving treatment, untreated LDL-C concentrations may be estimated using validated treatment adjustment factors to better assess the likelihood of inherited hypercholesterolemia. In addition to LDL-C, measurement of apolipoprotein B (apoB), non-high-density lipoprotein cholesterol (non-HDL-C), triglycerides, and lipoprotein(a) [Lp(a)] provides valuable information regarding the underlying dyslipidemic phenotype and residual cardiovascular risk. Elevated Lp(a) may contribute substantially to measured LDL-C concentrations and should be considered when interpreting lipid profiles in patients with suspected familial hypercholesterolemia.

### 4.3. Clinical Diagnostic Criteria

Several validated clinical scoring systems facilitate the diagnosis of familial hypercholesterolemia. The Dutch Lipid Clinic Network (DLCN) criteria remain the most widely used and incorporate LDL-C levels, family history, premature ASCVD, physical findings, and genetic testing results to estimate the probability of FH. The Simon Broome criteria similarly combine lipid levels with clinical and familial characteristics, whereas the MEDPED criteria rely primarily on age-specific LDL-C thresholds according to family history. Although these tools demonstrate good diagnostic performance, their sensitivity may be reduced in younger individuals, patients receiving intensive lipid-lowering therapy, or those lacking an informative family history.

### 4.4. Role and Limitations of Genetic Testing

Genetic testing has become an important component of the diagnostic evaluation by enabling identification of pathogenic variants in genes involved in LDL metabolism, including LDLR, APOB, PCSK9, and less frequently LDLRAP1 or APOE. Identification of a pathogenic or likely pathogenic variant confirms the molecular diagnosis, supports individualized risk assessment, and facilitates cascade screening among relatives.

Nevertheless, genetic testing should not be regarded as an absolute diagnostic gold standard. A substantial proportion of individuals fulfilling clinical criteria for familial hypercholesterolemia have no detectable pathogenic variant using currently available testing methods. These patients may have polygenic hypercholesterolemia, pathogenic variants in genes not routinely analyzed, or genetic mechanisms that remain incompletely characterized. Consequently, a negative genetic test does not exclude the diagnosis of familial hypercholesterolemia, and clinical judgment remains fundamental when evaluating patients with a highly suggestive phenotype.

### 4.5. Variants of Uncertain Significance

Increasing use of next-generation sequencing has led to the identification of numerous variants of uncertain significance (VUS). Such variants should not be considered diagnostic in isolation and should not guide predictive testing of family members. Their interpretation requires integration of clinical phenotype, family segregation analysis, population allele frequency, in silico prediction models, functional studies when available, and periodic re-evaluation according to the recommendations of the American College of Medical Genetics and Genomics (ACMG).

### 4.6. Cascade Screening

Cascade screening represents one of the most effective strategies for identifying previously undiagnosed individuals with familial hypercholesterolemia. Once an index case has been identified, first-degree relatives should undergo lipid profiling and, whenever a pathogenic familial variant has been identified, targeted genetic testing. Cascade screening enables diagnosis before the onset of clinical manifestations and has consistently demonstrated favorable cost-effectiveness while substantially improving long-term cardiovascular outcomes.

### 4.7. Emerging Biomarkers and Polygenic Risk Assessment

Additional biomarkers have expanded the diagnostic evaluation of inherited dyslipidemias. ApoB reflects the total number of circulating atherogenic lipoprotein particles, whereas non-HDL-C provides a comprehensive estimate of cholesterol contained within all atherogenic lipoproteins and is particularly informative in patients with hypertriglyceridemia. Measurement of Lp(a) is recommended at least once during adulthood because markedly elevated concentrations independently increase ASCVD risk and may modify therapeutic decision-making.

Polygenic risk scores (PRSs) have emerged as promising tools for explaining hypercholesterolemia in patients without identifiable monogenic mutations and may contribute to refined cardiovascular risk stratification. However, their routine clinical implementation remains limited by methodological heterogeneity, reduced predictive performance across diverse ancestral populations, and insufficient prospective validation. Accordingly, current evidence supports the use of PRS as complementary rather than replacement tools alongside established clinical and genetic assessment.

### 4.8. Vascular Imaging for Risk Stratification

Although vascular imaging is not required to establish the diagnosis of familial hypercholesterolemia, it provides important information regarding the burden of subclinical atherosclerosis and may improve cardiovascular risk stratification. Coronary artery calcium scoring, carotid ultrasonography, coronary computed tomography angiography, and selected functional imaging techniques may assist in identifying patients at particularly high cardiovascular risk and in guiding treatment intensity, especially in individuals with intermediate clinical risk or uncertain phenotypic expression.

### 4.9. Practical Diagnostic Approach

**Figure 4:**
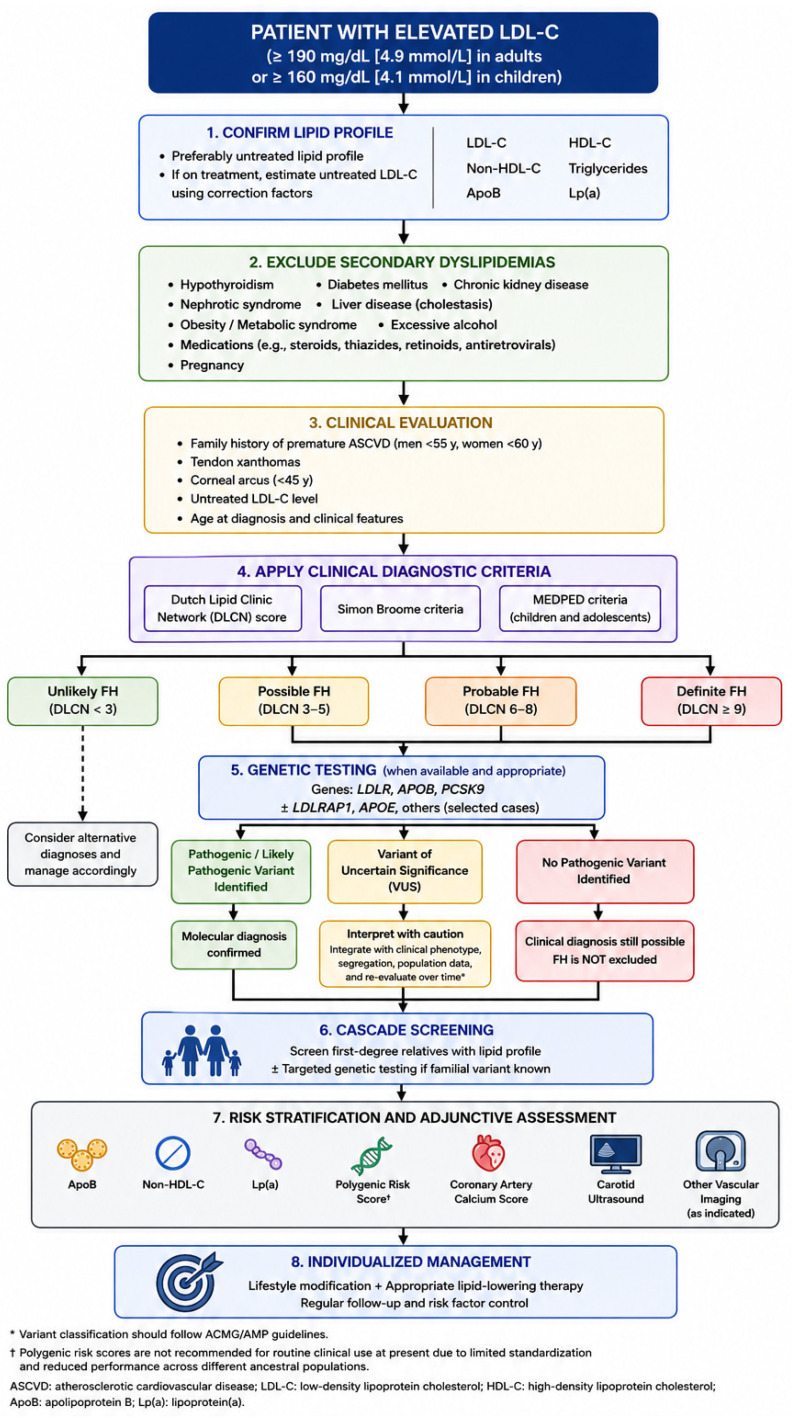
Clinical workflow for the diagnosis and risk stratification of familial hypercholesterolemia.

The diagnosis of familial hypercholesterolemia should be regarded as a stepwise process integrating clinical phenotype, exclusion of secondary causes, lipid profile interpretation, validated clinical scoring systems, genetic testing when appropriate, family cascade screening, and complementary biomarkers or vascular imaging. Such an integrated approach maximizes diagnostic accuracy while recognizing that inherited dyslipidemias represent a heterogeneous spectrum in which both monogenic and polygenic mechanisms contribute to disease expression (Figure 4).

## 5. Emerging RNA-Based and Gene Editing Therapies

Recent advances in RNA-based therapeutics and gene editing technologies are rapidly expanding the therapeutic landscape of inherited dyslipidemias beyond conventional lipid-lowering agents. Small interfering RNA (siRNA) therapies targeting PCSK9, including inclisiran, have demonstrated sustained LDL-C reduction with a convenient twice-yearly maintenance dosing schedule following the initial loading phase, potentially improving long-term treatment adherence. In parallel, several RNA-based therapies directed against lipoprotein(a) [Lp(a)] synthesis—including the antisense oligonucleotide pelacarsen and siRNA agents such as olpasiran, lepodisiran, and zerlasiran—have demonstrated substantial reductions in circulating Lp(a) concentrations in early-phase clinical trials and are currently being evaluated in large cardiovascular outcome studies.

Gene editing technologies represent one of the most promising future directions in the management of inherited dyslipidemias. In particular, CRISPR-based approaches targeting PCSK9 have shown encouraging preliminary clinical results, raising the possibility of durable LDL-C reduction following a single therapeutic intervention. Although these innovative strategies remain investigational and require further evaluation regarding long-term efficacy and safety, they illustrate the transition toward precision medicine aimed at correcting the underlying molecular defects responsible for inherited lipid disorders.

## 6. Current Challenges and Future Perspectives

Despite remarkable advances in lipid-lowering therapy, several important challenges remain. Long-term safety data for recently introduced therapies, particularly in pediatric populations, are still limited, and continued post-marketing surveillance will be essential. In addition, treatment adherence remains a major determinant of therapeutic success in lifelong conditions such as familial hypercholesterolemia. The high cost of novel biological therapies, unequal access to specialized care, and reimbursement restrictions continue to limit their widespread implementation in many healthcare systems.

Future management of inherited dyslipidemias is expected to increasingly integrate molecular diagnosis, individualized cardiovascular risk assessment, and precision medicine strategies to optimize therapeutic decision-making and improve long-term outcomes. Furthermore, advances in multi-omics technologies—including lipidomics, metabolomics, proteomics, and single-cell transcriptomics—together with machine learning and artificial intelligence-based predictive models, are expected to refine disease phenotyping, identify novel therapeutic targets, and facilitate more personalized prevention and treatment strategies. Continued integration of these emerging technologies into clinical practice may further improve risk stratification and enable a more individualized approach to the management of familial hypercholesterolemia and familial combined hyperlipidemia.

## 7. Conclusions

Primary hyperlipoproteinemias are no longer viewed solely as disorders defined by isolated lipid elevations but rather as dynamic, genetically and metabolically complex conditions that require an integrated, life-course approach to diagnosis and management. The transition from traditional phenotypic classification toward molecular characterization has profoundly refined our understanding of disease heterogeneity, cardiovascular risk stratification, and therapeutic decision-making. Monogenic familial hypercholesterolemia (FH) remains a paradigmatic model of causality between lifelong LDL-C exposure and premature atherosclerotic cardiovascular disease (ASCVD). Variants in LDLR, APOB, PCSK9, and rare APOE mutations demonstrate how disruption of receptor-mediated LDL clearance leads to markedly elevated LDL-C from birth, resulting in a cumulative “cholesterol burden” that directly determines vascular injury. The distinction between heterozygous and homozygous FH is clinically critical, as the latter requires immediate, specialized, and often multimodal therapy to prevent early cardiovascular events. Importantly, genetic confirmation not only solidifies diagnosis but facilitates cascade screening and individualized therapeutic intensity. However, a substantial proportion of individuals with a clinical FH phenotype do not harbor a single pathogenic mutation. In these cases, polygenic predisposition—driven by the additive effect of multiple LDL-C-raising variants—accounts for much of the observed hypercholesterolemia. Polygenic risk scores provide valuable insight into phenotype severity and cardiovascular risk modulation, even among carriers of monogenic variants. This underscores that hypercholesterolemia exists along a genetic continuum rather than within rigid diagnostic categories. Lipoprotein(a) further refines this landscape, acting as an independent and genetically determined amplifier of cardiovascular risk. Elevated Lp(a), particularly when combined with high LDL-C, substantially augments ASCVD risk and may partially explain variability within FH cohorts. Its measurement is therefore essential for comprehensive risk assessment and therapeutic planning.

Familial combined hyperlipidemia (FCHL) exemplifies another dimension of inherited dyslipidemia—one characterized by polygenic architecture, hepatic VLDL overproduction, insulin resistance, and metabolic flexibility. The marked intrafamilial variability and dynamic lipid phenotype reflect the interaction between genetic susceptibility and environmental factors such as adiposity and hepatic steatosis. In such patients, reliance solely on LDL-C may underestimate risk; non-HDL cholesterol and apolipoprotein B offer more accurate reflection of atherogenic particle burden. Therapeutically, advances in lipid-lowering strategies—from statins and ezetimibe to PCSK9 inhibitors, ANGPTL3 inhibition, and emerging gene-silencing technologies—have transformed management paradigms. Yet, despite increasingly effective pharmacologic tools, residual risk persists, largely attributable to delayed diagnosis and cumulative exposure to elevated atherogenic lipoproteins. Early detection of FH, particularly in childhood, remains the single most impactful intervention. Long-term observational data clearly demonstrate that treatment initiated in youth dramatically reduces cardiovascular morbidity and mortality.

Ultimately, optimal management of primary hyperlipoproteinemias requires a precision-based framework integrating genetic testing, polygenic risk assessment, lipoprotein phenotyping, imaging of subclinical atherosclerosis, and consideration of metabolic comorbidities. Future research should focus on refining lifetime LDL-C exposure models, clarifying the role of vascular imaging in treatment monitoring, and expanding equitable access to advanced therapies worldwide.

In conclusion, the convergence of molecular genetics, metabolic insight, and therapeutic innovation is reshaping the field of inherited lipid disorders. A shift from static lipid thresholds toward individualized lifetime risk assessment represents the next frontier in preventing cardiovascular disease in patients with primary hyperlipoproteinemias.

## Figures and Tables

**Table 1:** Fredrickson Classification of Primary Hyperlipoproteinemias.

Type	Elevated Lipoprotein(s)	Main Lipid Abnormality	Typical Clinical Association
**Type I**	Chylomicrons	Severe hypertriglyceridemia	Recurrent pancreatitis; rare; usually LPL or ApoC-II deficiency
**Type IIa**	LDL	Isolated hypercholesterolemia	Premature ASCVD (e.g., familial hypercholesterolemia)
**Type IIb**	LDL + VLDL	Combined hyperlipidemia (↑ LDL and TG)	Familial combined hyperlipidemia; high ASCVD risk
**Type III**	IDL (remnant particles)	Elevated cholesterol and triglycerides	Dysbetalipoproteinemia; palmar xanthomas; premature ASCVD
**Type IV**	VLDL	Hypertriglyceridemia	Associated with insulin resistance, obesity, T2DM
**Type V**	Chylomicrons + VLDL	Severe mixed hypertriglyceridemia	High pancreatitis risk

**Table 2:** Genetic Architecture of Familial Hypercholesterolemia (FH) Phenotype.

Category	Gene/Mechanism	Molecular Defect	Effect on LDL Metabolism	Phenotypic Features	Relative CHD Risk	Clinical Implications
**Monogenic FH (Autosomal Dominant)**	LDLR	Loss-of-function variants (binding, internalization, recycling defects)	Reduced hepatic LDL receptor activity → markedly decreased LDL-C clearance	Typically severe LDL-C elevation from birth; severity depends on residual receptor function	Very high (due to lifelong LDL-C exposure)	Requires early and intensive lipid-lowering therapy
	APOB (ApoB-100)	Missense variants impairing LDL receptor binding (e.g., ligand-defective ApoB)	Reduced affinity of LDL particle for LDL receptor → impaired clearance	Similar to LDLR-FH but often milder	High	Treatment similar to LDLR-FH; phenotype influenced by binding impairment
	PCSK9 (Gain-of-function)	Increased LDL receptor degradation	Reduced receptor recycling → increased LDL-C	Variable severity; may be severe	High to very high	Strong response to PCSK9 inhibition
	APOE (rare variants such as p.Leu167del)	Impaired ApoE-mediated lipoprotein clearance; reduced LDLR expression	Decreased hepatic clearance of ApoE-containing lipoproteins	FH-like phenotype; rare	Elevated	Rare cause; requires genetic confirmation
**LPA Contribution to FH Phenotype**	LPA gene (kringle IV-2 repeat variation, regulatory variants)	Increased production of Lp(a)	Elevated Lp(a); independent of LDL receptor pathway	May coexist with FH; higher median Lp(a) in clinical FH	Independently increases CHD risk; synergistic with high LDL-C	Lp(a) measurement important for risk stratification
**Previously Proposed but Refuted Gene**	STAP1	Initially suspected; no confirmed functional link	No proven effect on LDL metabolism	Not causative	Not applicable	Example of need for rigorous validation
**Polygenic Hypercholesterolemia**	Multiple common LDL-C–raising SNPs	Cumulative small-effect variants (high LDL-C genetic risk score)	Modest but sustained increase in LDL-C	Often mutation-negative clinical FH; LDL-C elevation variable	Moderate to high (lower than monogenic FH at same LDL-C level)	Requires lipid-lowering therapy; intensity guided by overall risk
**Monogenic FH + High Polygenic Burden**	Monogenic variant plus elevated LDL-C GRS	Additive genetic effects	Higher LDL-C and/or greater CHD susceptibility	More severe phenotype than mutation alone	Higher than monogenic FH with low GRS	May justify intensified treatment
**Key Pathophysiological Distinction**	Lifelong LDL-C burden	Early-onset vs. gradual LDL-C elevation	Cumulative exposure determines atherosclerosis risk	Monogenic: high from birth; Polygenic: gradual increase	Monogenic > Polygenic (at same measured LDL-C)	Early detection critical in monogenic FH

**Table 3:** Comparison of Dutch Lipid Clinic Network (DLCN) and Simon–Broome Criteria.

Feature	Dutch Lipid Clinic Network (DLCN)	Simon–Broome Criteria
**Approach**	Quantitative point-based scoring system	Categorical diagnostic criteria
**LDL-C Threshold**	Graded scoring based on untreated LDL-C levels	Adults: Total cholesterol ≥7.5 mmol/L (≈290 mg/dL) or LDL-C ≥4.9 mmol/L (≈190 mg/dL); Children: Total cholesterol ≥6.7 mmol/L (≈260 mg/dL) or LDL-C ≥4.0 mmol/L (≈155 mg/dL)
**Family History**	Points assigned for premature ASCVD or elevated LDL-C in first-degree relatives	Requires family history of premature CAD or elevated cholesterol for “possible FH”
**Clinical Signs**	Tendon xanthomas (6 points); arcus cornealis <45 years (4 points)	Tendon xanthomas in patient or first-/second-degree relative required for “definite FH”
**Genetic Testing**	Functional mutation scores 8 points	Pathogenic mutation confirms “definite FH”
**Diagnostic Categories**	>8: Definite FH; 6–8: Probable FH; 3–5: Possible FH; <3: Unlikely	Definite FH: Elevated cholesterol + tendon xanthomas or pathogenic mutation; Possible FH: Elevated cholesterol + family history
**Strength**	More nuanced risk stratification through scoring	Simpler, clinically practical classification
**Limitation**	Requires detailed data for accurate scoring	Less granular; may miss intermediate-risk individuals

**Table 4:** Lipid-Lowering Therapies Approved for or Under Investigation in Pediatric Familial Hypercholesterolemia (FH).

Class/Drug(s)	Mechanism of Action	Administration	Effect on Lipoproteins	Tolerability/Main Adverse Effects
**Statins** Atorvastatin, Fluvastatin, Lovastatin, Pitavastatin, Pravastatin, Rosuvastatin, Simvastatin				
	HMG-CoA reductase inhibitors → decrease hepatic cholesterol synthesis → upregulation of LDL receptors → enhanced LDL clearance	Oral tablet, once daily	LDL-C reduction ≈20–50% (dose- and drug-dependent; influenced by LDL receptor function); modest HDL-C increase; triglyceride reduction variable	Generally well tolerated; may cause elevated hepatic transaminases, myalgia/myopathy, rare rhabdomyolysis; small increased risk of incident diabetes; potential drug interactions
**Cholesterol Absorption Inhibitor** **Ezetimibe**				
	**Mechanism**	**Administration**	**Effect**	**Adverse Effects**
	Inhibits NPC1L1 transporter in small intestine → reduces cholesterol absorption	Oral tablet, once daily	LDL-C reduction ≈15–20%	Well tolerated; typically adjunct to statins; risk of hepatic transaminase elevation or myopathy when combined with statins; arthralgia, diarrhea, sinusitis, fatigue, upper respiratory infection
**PCSK9 Pathway Inhibitors** **Monoclonal Antibodies** Alirocumab, Evolocumab				
	**Mechanism**	**Administration**	**Effect**	**Adverse Effects**
	Bind circulating PCSK9 → prevent LDL receptor degradation → increase LDL receptor expression and LDL clearance	Subcutaneous injection every 2–4 weeks	LDL-C reduction ≈30–40%	Generally well tolerated; injection site reactions; arthralgia, back pain, nasopharyngitis, upper respiratory infection, nausea
**Inclisiran (siRNA against PCSK9)**				
	**Mechanism**	**Administration**	**Effect**	**Adverse Effects**
	Small interfering RNA inhibits hepatic PCSK9 synthesis → increases LDL receptor expression	Subcutaneous injection: 3 doses in first year, then every 6 months	LDL-C reduction ≈40–60% in adults (not yet approved for pediatric use)	Well tolerated; injection site reactions
**ATP–Citrate Lyase Inhibitor** **Bempedoic Acid**				
	**Mechanism**	**Administration**	**Effect**	**Adverse Effects**
	Inhibits ATP–citrate lyase → reduces cholesterol synthesis → increases LDL receptor expression	Oral tablet, once daily	LDL-C reduction ≈17–28% in adults (not yet approved for pediatric use)	Generally well tolerated; may cause muscle spasm, back or extremity pain, hyperuricemia, gout, cholelithiasis, renal impairment, abdominal discomfort, anemia, transaminitis, rare tendon rupture
**Bile Acid Sequestrants** Cholestyramine, Colestipol, Colesevelam				
	**Mechanism**	**Administration**	**Effect**	**Adverse Effects**
	Bind bile acids in intestine → prevent enterohepatic reabsorption → increase bile acid synthesis → enhance LDL uptake	Oral powder or tablets, daily	LDL-C reduction ≈10–20%; may increase triglycerides	Gastrointestinal intolerance common (bloating, constipation); cholestyramine poorly palatable; colestipol requires multiple large tablets; colesevelam better tolerated; risk of fat-soluble vitamin deficiency; drug interactions
**Therapies Specifically for Homozygous FH (HoFH)** **Evinacumab**				
	**Mechanism**	**Administration**	**Effect**	**Adverse Effects**
	ANGPTL3 inhibition → increases lipoprotein lipase and endothelial lipase activity	Intravenous infusion every 4 weeks	LDL-C reduction ≈50%; reduces non-HDL-C, HDL-C, and lipoprotein(a)	Well tolerated; infusion reactions; nasopharyngitis, rhinorrhea, flu-like illness, dizziness, fatigue, nausea; reserved for HoFH
**Lomitapide**				
	**Mechanism**	**Administration**	**Effect**	**Adverse Effects**
	Inhibits microsomal triglyceride transfer protein (MTP) → decreases assembly of ApoB-containing lipoproteins	Oral tablet, daily	LDL-C reduction ≈40–60% (dose-dependent); pediatric HoFH study showed 54% reduction; not yet approved for pediatric use	Variable tolerability; strict low-fat diet required; gastrointestinal symptoms; hepatic transaminase elevation; hepatic steatosis; nutritional deficiencies; reserved for HoFH

Abbreviations: ATP—adenosine triphosphate. FH—familial hypercholesterolemia. HoFH—homozygous familial hypercholesterolemia. HDL—high-density lipoprotein. HMG-CoA—3-hydroxy-3-methylglutaryl–coenzyme A. LDL—low-density lipoprotein. LDL-C—low-density lipoprotein cholesterol. MTP—microsomal triglyceride transfer protein NPC1L1—Niemann–Pick C1-like protein 1. PCSK9—proprotein convertase subtilisin/kexin type 9.

**Table 5:** Overview of Familial Combined Hyperlipidemia (FCHL): Genetic and Metabolic Characteristics.

Section	Key Elements	Summary of Information
Historical Background	First description (1973)	Initially described by Goldstein et al. [26]; similar reports by Rose et al. [27] and Nikkilä and Aro [28] in the same year.
	Early inheritance model	Initially considered an autosomal dominant lipid disorder associated with premature atherosclerosis.
	Epidemiological validation	Later studies confirmed its high prevalence and cardiovascular risk.
Genetic Basis	Early hypothesis	Extensive search for a single causative gene was unsuccessful.
	Current understanding	Recognized as a polygenic disorder involving multiple genetic variants.
	Genetic contributors	Combination of common susceptibility alleles and rare variants with incomplete penetrance.
	Phenotypic heterogeneity	Different lipid phenotypes (hypercholesterolemia, hypertriglyceridemia, mixed dyslipidemia) may coexist within the same family.
Clinical Risk Profile	Cardiovascular risk	Significantly increased risk of premature ASCVD.
	Metabolic comorbidities	Increased predisposition to type 2 diabetes mellitus and steatotic liver disease.
Core Metabolic Abnormalities	Hepatic VLDL overproduction	One of the earliest and most consistently described abnormalities.
	Insulin resistance	Strongly associated and considered a central metabolic feature.
	Chylomicron remnant clearance	Delayed clearance contributing to postprandial dyslipidemia.
	Endothelial lipoprotein retention	Increased levels of atherogenic lipoproteins bound to vascular endothelium.
Pathophysiological Mechanisms	Free fatty acid (FFA) flux	Impaired peripheral FFA uptake may increase hepatic FFA delivery, stimulating VLDL synthesis.
	Supporting evidence	Small in vivo study showed exaggerated postprandial ketone body production, suggesting enhanced hepatic fatty acid flux.
Complement System Involvement	Acylation-stimulating protein (ASP)	Impaired ASP function described in hyperapoB individuals; ASP later identified as C3adesArg.
	Complement C3	Linked to triglyceride-rich lipoprotein metabolism and postprandial lipid handling.
	C5L2 mutation	Mutation in ASP receptor (C5L2) identified in one FCHL family, suggesting complement pathway involvement.
	Current status	Complement system contribution suggested but pathogenetic role remains unclear.

## Data Availability

No new data were created or analyzed in this study. Data sharing is not applicable to this article.
